# Genetic risk and atrial fibrillation in patients with heart failure

**DOI:** 10.1002/ejhf.1735

**Published:** 2020-01-09

**Authors:** Mariëlle Kloosterman, Bernadet T. Santema, Carolina Roselli, Christopher P. Nelson, Andrea Koekemoer, Simon. P.R. Romaine, Isabelle C. Van Gelder, Carolyn S.P. Lam, Vicente A. Artola, Chim C. Lang, Leon L. Ng, Marco Metra, Stefan Anker, Gerasimos Filippatos, Kenneth Dickstein, Piotr Ponikowski, Pim van der Harst, Peter van der Meer, Dirk J. van Veldhuisen, Emelia J. Benjamin, Adriaan A. Voors, Nilesh J. Samani, Michiel Rienstra

**Affiliations:** ^1^ Department of Cardiology University of Groningen, University Medical Center Groningen Groningen The Netherlands; ^2^ Cardiovascular Disease Initiative The Broad Institute of MIT and Harvard Cambridge MA USA; ^3^ Department of Cardiovascular Sciences University of Leicester and NIHR Leicester Biomedical Research Centre Leicester UK; ^4^ National Heart Centre Singapore Singapore; ^5^ School of Medicine Centre for Cardiovascular and Lung Biology, Division of Molecular and Clinical Medicine University of Dundee, Ninewells Hospital & Medical School Dundee UK; ^6^ Institute of Cardiology, Department of Medical and Surgical Specialties, Radiological Sciences and Public Health University of Brescia Brescia Italy; ^7^ Department of Cardiology (CVK) Berlin Institute of Health Center for Regenerative Therapies (BCRT); German Centre for Cardiovascular Research (DZHK) partner site Berlin; Charité Universitätsmedizin Berlin Berlin Germany; ^8^ Athens University Hospital Athens Greece; ^9^ University of Bergen, Stavanger University Hospital Bergen Norway; ^10^ Department for Heart Disease Centre for Heart Disease, University Hospital, Medical University Wroclaw Poland; ^11^ Department of Medicine, Boston University School of Medicine, Department of Epidemiology Boston University School of Public Health Boston MA USA

**Keywords:** Atrial fibrillation, Heart failure, Genetic association studies, Single nucleotide polymorphism, Risk factors

## Abstract

**Aims:**

To study the association between an atrial fibrillation (AF) genetic risk score with prevalent AF and all‐cause mortality in patients with heart failure.

**Methods and results:**

An AF genetic risk score was calculated in 3759 European ancestry individuals (1783 with sinus rhythm, 1976 with AF) from the BIOlogy Study to TAilored Treatment in Chronic Heart Failure (BIOSTAT‐CHF) by summing 97 single nucleotide polymorphism (SNP) alleles (ranging from 0–2) weighted by the natural logarithm of the relative SNP risk from the latest AF genome‐wide association study. Further, we assessed AF risk variance explained by additive SNP variation, and performance of clinical or genetic risk factors, and the combination in classifying AF prevalence. AF was classified as AF or atrial flutter (AFL) at baseline electrocardiogram and/or a history of AF or AFL. The genetic risk score was associated with AF after multivariable adjustment. Odds ratio for AF prevalence per 1‐unit increase genetic risk score was 2.12 (95% confidence interval 1.84–2.45, *P* = 2.15 × 10^−24^) in the total cohort, 2.08 (1.72–2.50, *P* = 1.30 × 10^−14^) in heart failure with reduced ejection fraction (HFrEF) and 2.02 (1.37–2.99, *P* = 4.37 × 10^−4^) in heart failure with preserved ejection fraction (HFpEF). AF‐associated loci explained 22.9% of overall AF SNP heritability. Addition of the genetic risk score to clinical risk factors increased the C‐index by 2.2% to 0.721.

**Conclusions:**

The AF genetic risk score was associated with increased AF prevalence in HFrEF and HFpEF. Genetic variation accounted for 22.9% of overall AF SNP heritability. Addition of genetic risk to clinical risk improved model performance in classifying AF prevalence.

## Introduction

Atrial fibrillation (AF) is the most common cardiac arrhythmia and is highly prevalent in patients with heart failure.^1^, ^2^, ^3^ The co‐existence of these conditions can be expected by virtue of their prevalence alone: the lifetime risk of developing AF is about one in three in individuals of European ancestry and one in five in individuals of African ancestry,^4^, ^5^, ^6^ and after age 45 the lifetime risk of heart failure ranges between 20–45%.^7^

Furthermore, both conditions have many shared risk factors which makes their co‐existence more likely.^8^, ^9^ Additionally, a reciprocal relation between both conditions seems to exist, but regardless of which condition occurs first, the concomitant presence of both AF and heart failure is associated with substantially increased risks of mortality.^2^, ^3^

Atrial fibrillation is common in heart failure and prevalence of the arrhythmia increases with heart failure severity, but little is known about the mechanisms that underlie AF onset in heart failure patients.^10^, ^11^ Genetic factors could theoretically explain, at least partly, the increased risk of AF in patients with heart failure.^12^ But heritability of AF is complex; in a recent study, 97 genome‐wide susceptibility loci for AF were identified, and the proportion of heritability explained by the loci in individuals of European ancestry was 42%.^13^ Prevalence estimates of heart failure in population‐based biobanks and case‐referent studies used for AF genome‐wide association studies (GWAS) is limited, and it remains unclear whether individuals with AF in the context of heart failure share a similar genetic susceptibility to the arrhythmia.

We aimed to study the association between a genetic risk score based on 97 lead single nucleotide polymorphisms (SNPs)^13^ with prevalent AF and all‐cause mortality in a large sample of patients with heart failure included in The BIOlogy Study to TAilored Treatment in Chronic Heart Failure (BIOSTAT‐CHF) . Further, we assessed the variance in AF prevalence explained by additive SNP variation (SNP heritability), and determined the discriminatory accuracy of clinical risk factors, genetic risk factors, and the combination in classifying AF prevalence.

## Methods

### Study population

The prospective, observational, international BIOSTAT‐CHF study included 2516 patients with heart failure from 11 European countries between December 2010 and December 2012. Another 1738 patients from Scotland were included in a validation cohort between October 2010 and April 2014. The rationale, design, and primary results have been previously published.^14^ Briefly, the majority of patients were hospitalized for acute heart failure, and the remainder presented with worsening signs and/or symptoms of heart failure at outpatient clinics. Patients had to have objective evidence of cardiac dysfunction documented either by left ventricular ejection fraction (LVEF) of ≤40%, previous heart failure hospitalization, or plasma concentrations of B‐type natriuretic peptide (BNP) and/or N‐terminal pro‐B‐type natriuretic peptide (NT‐proBNP) >400 pg/mL or > 2000 pg/mL, respectively. According to study design, all patients used diuretics but were not on optimal, evidence‐based medical therapy of angiotensin‐converting enzyme inhibitors/angiotensin receptor blockers and, or beta‐blockers. After inclusion patients were extensively phenotyped and genotyped, underwent physical examination and quality of life measurements, and plasma, serum, and urine samples were collected for analysis. During the first 3 months of follow‐up, medication was optimized. The study complies with the Declaration of Helsinki, medical ethics committee of participating centres approved the study, and all patients provided written informed consent before inclusion.

### Patient selection

**Figure 1:**
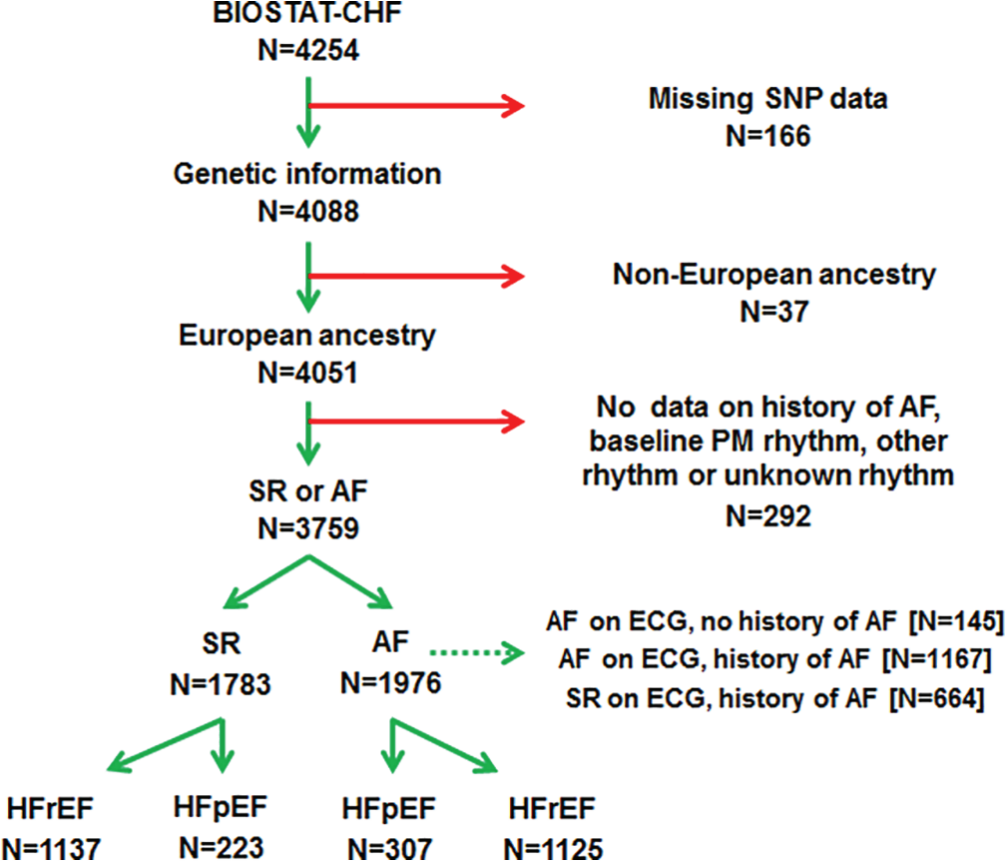
Flowchart of the final BIOSTAT‐CHF study population. AF, atrial fibrillation; ECG, electrocardiogram; HFpEF, heart failure with preserved ejection fraction; HFrEF, heart failure with reduced ejection fraction; PM, pacemaker; SNP, single nucleotide polymorphism; SR, sinus rhythm.

For the current analysis the BIOSTAT‐CHF index cohort (*n* = 2516) and validation cohort (*n* = 1738) were combined to achieve a larger set of patients (*n* = 4254). Patients with no blood samples available for genotyping (*n* = 166), self‐reported non‐European ancestry (*n* = 37), and pacemaker rhythm or missing variables that prohibited rhythm classification (*n* = 292) were excluded (*Figure* 1).

### Atrial fibrillation prevalence and all‐cause mortality

Atrial fibrillation prevalence was defined as clinical history of AF or atrial flutter (AFL) and/or AF(L) on baseline electrocardiogram (ECG). Patients were regarded as having sinus rhythm if they had no history of AF and sinus rhythm on baseline ECG. Incident AF was not captured during follow‐up.

After the optimization (3 months) and maintenance phase (6 months),^14^ patients were followed by standard clinical follow‐up or telephone contact with 6‐month intervals. Follow‐up ended on April 1st 2015. Median follow‐up duration was approximately 21 months. During follow‐up all deaths and hospitalizations were recorded. For the current analysis, all‐cause mortality was assessed.

### Genotyping in BIOSTAT‐CHF

The two cohorts were processed, genotyped, QC'd and imputed independently, using the same protocols. Genotyping of all patients from both BIOSTAT‐CHF cohorts was performed using the Affymetrix Axiom Genome‐Wide UKB WCSG genotyping array. Sample level QC was performed for X chromosome homozygosity (sex mismatch) and identity by descent (IBD) estimates (relatedness and duplicates). Prior to imputation, variants were removed if their call rate was <95% for variants with minor allele frequency (MAF) ≥5%, or < 99% for variants with MAF <5%, or had a Hardy–Weinberg equilibrium *P* < 1x10^−6^. Imputation was performed using SHAPEIT2^15^ and IMPUTE2^16^ with the phase 3 release 1000G reference panel.^17^

### Genetic analysis

#### Atrial fibrillation genetic risk score

Genotypes of 97 SNPs associated with AF risk in the latest published GWAS^13^ with significance thresholds of *P* < 1 × 10^−8^ were used to calculate an individual patient AF genetic risk score by summing the dosage of each AF risk allele in BIOSTAT‐CHF (ranging from 0–2) weighted by the natural logarithm of the relative risk for each SNP. Weights were determined by the latest AF GWAS^13^ (online supplementary *Table S1*). The SNP rs465276 was not available in BIOSTAT‐CHF and was substituted with a proxy (rs361834, r^2^ = 0.91, based on pairwise linkage disequilibrium from European ancestry samples in the Broad AF study^13^). All SNPs had an INFO score >0.4 and a Hardy–Weinberg equilibrium *P* > 1 × 10^−6^. AF genetic risk scores were calculated using PLINK v2.00.^18^

#### Proportion of heritability explained

We assessed the proportion of AF phenotypic variance explained by additive genetic variation, otherwise referred to as SNP heritability (h^2^_g_). h^2^_g_ was calculated with the software BOLT‐LMM v2.3.2.^19^ The AF loci were defined as a region of 1 Mb (±500 kb) around each of the 97 reported sentinel variants from the latest AF GWAS analysis.^13^ We used the imputed genotype data, filtered the variants for imputation quality >0.8, as calculated by QCTOOL v2,^20^ hard‐called the genotypes with a genotype probability threshold >0.9 with PLINK v2.00,^18^ and combined the overlapping variants that remained from the index and validation cohort of BIOSTAT‐CHF. Additional filtering removed variants with MAF <1% and variant call rate missingness >0.5%. We then applied one round of pruning with the settings – indep‐pairwise 50 5 0.9 in PLINK. The heritability calculation was performed on the remaining 806130 variants. We included age, sex, and the first five principal components as covariates. The observed heritability estimates were converted to the liability scale following equation 17 from Lee *et al*.^21^ and using the AF prevalence in the BIOSTAT‐CHF cohorts (AF prevalence of 53%) as disease prevalence in a heart failure population.

### Statistical analyses

Normally distributed variables are depicted as means ± standard deviation and non‐normally distributed variables as median with the first and third quartile (Q1, Q3). Categorical variables are presented as numbers with percentages. Multivariable logistic regression models were used to examine whether a genetic risk score build of 97 AF genetic loci was associated with AF prevalence. Model 1 was adjusted for age, sex, and the first 10 principal components of ancestry. Model 2 was adjusted for clinical AF risk factors from the CHARGE‐AF risk model,^22^ a model aimed to predict future risk of AF. Variables include: age, height, weight, systolic and diastolic blood pressure, current smoking, hypertension as a proxy for antihypertensive treatment, diabetes, myocardial infarction, and the first 10 principal components of ancestry. The CHARGE‐AF risk model variables heart failure and race were not included since our population consists of European ancestry patients with heart failure. A total of 96 patients had missing values and were excluded. We calculated the area under the receiver operating curve (AUC) in logistic regression models for AF prevalence. All calculations included the first 10 principal components and were performed in R using the package *pROC* 23 to calculate the AUC and the 95% confidence intervals (CI) with the DeLong method. Cox proportional hazard analysis was performed to determine hazard ratios (HR) with 95% CI for the genetic risk score and all‐cause mortality. All HR were adjusted for covariates of the CHARGE‐AF risk model. The Cox proportional hazards assumption was assessed by visually inspecting plots of Schoenfeld residuals against time, which showed no proportionality violation (i.e. the plots showed random patterns of residuals against time). Interaction testing was performed to determine whether the effect of the genetic risk score differed between the heart failure phenotypes, with regard to AF prevalence and all‐cause mortality risk. Secondary analyses were performed in subgroups based on LVEF: LVEF <40% and LVEF ≥50%, respectively, heart failure with reduced ejection fraction (HFrEF) and heart failure with preserved ejection fraction (HFpEF). Patients with a mid‐range ejection fraction (LVEF 40–49%) or missing LVEF data were not assessed separately. Analyses were performed using IBM SPSS Statistics version 23. The a priori significance threshold for all analyses was *P* < 0.05 using 2‐sided tests.

## Results

### Patient characteristics

An overview of the cohort is shown in *Table* 1. A total of 3759 European ancestry individuals from BIOSTAT‐CHF were included, of whom 1976 (53%) had prevalent AF. Mean age was 72.8 ± 11.5, 30% were women. These patients were further stratified in 2262 HFrEF patients, of whom 1137 (50.3%) were in sinus rhythm and 1125 (49.7%) had AF; and 530 HFpEF patients, of whom 223 (42%) were in sinus rhythm and 307 (58%) had AF (*Figure* 1). Overall, patients with AF were older (75.0 ± 10.2 vs. 70.3 ± 12.3 years), more often men (73% vs. 67%), and had a higher body mass index (28.7 ± 5.9 vs. 28.0 ± 5.9 kg/m^2^). AF patients more often had renal disease (38% vs. 29%), but less often had coronary artery disease (43% vs. 54%) (all *P* < 0.001).

**Table 1:** Baseline characteristics

	Overall (*n* = 3759)	AF (*n* = 1976, 53%)	SR (*n* = 1783, 47%)	*P*‐value
Demographics				
Age, years	72.8 ± 11.5	75.0 ± 10.2	70.3 ± 12.3	<0.001
Women, *n* (%)	1128 (30)	537 (27)	591 (33)	<0.001
NYHA class I/II/III/IV, %	6/43/36/7	5/46/41/8	8/47/37/8	0.001
Clinical variables				
BMI, kg/m^2^	28.3 ± 5.9	28.7 ± 5.9	28.0 ± 5.9	<0.001
Blood pressure, mmHg
Systolic	125 ± 22	124 ± 21	127 ± 23	0.002
Diastolic	73 ± 14	73 ± 14	72 ± 13	0.01
Heart rate, bpm	78 ± 19	80 ± 21	75 ± 16	<0.001
Medical history, *n* (%)				
Coronary artery disease^a^	1814 (48)	856 (43)	958 (54)	<0.001
Hypertension	2295 (61)	1221 (62)	1074 (60)	0.32
Diabetes mellitus	1218 (32)	657 (33)	561 (31)	0.25
Renal disease^a^	1276 (34)	757 (38)	519 (29)	<0.001
Echocardiographic data				
LVEF, %	35 ± 13	36 ± 13	34 ± 13	<0.001
HFrEF^b^, *n* (%)	2262 (60)	1125 (57)	1137 (64)	<0.001
HFpEF^c^, *n* (%)	530 (14)	307 (16)	223 (13)	<0.001
Laboratory data				
NT‐proBNP, ng/L, median (IQR)	2096 (825–4861)	2537 (1128–5122)	1588 (515–4510)	<0.001
Medications, *n* (%)				
ACEi/ARB	2681 (71)	1370 (69)	1311 (74)	0.005
Beta‐blocker	2410 (64)	1307 (66)	1103 (62)	0.18
MRA	1670 (44)	872 (44)	798 (45)	0.37
Diuretics	3735 (99)	1960 (99)	1775 (99)	0.01

ACEi, angiotensin‐converting enzyme inhibitor; AF, atrial fibrillation; ARB, angiotensin receptor blocker; BMI, body mass index; HFpEF, heart failure with preserved ejection fraction; HFrEF, heart failure with reduced ejection fraction; IQR, interquartile range; LVEF, left ventricular ejection fraction; MRA, mineralocorticoid receptor antagonist; NT‐proBNP, N‐terminal pro‐B‐type natriuretic peptide; NYHA, New York Heart Association; SR, sinus rhythm.

a Coronary artery disease defined as: previous myocardial infarction, percutaneous coronary intervention and/or coronary artery bypass graft. Renal disease defined as estimated glomerular filtration rate < 60 mL/min/1.73 m^2^.

b HFrEF defined as LVEF <40%.

c HFpEF defined as LVEF ≥50%.

### Genetic risk score and atrial fibrillation prevalence

**Figure 2:**
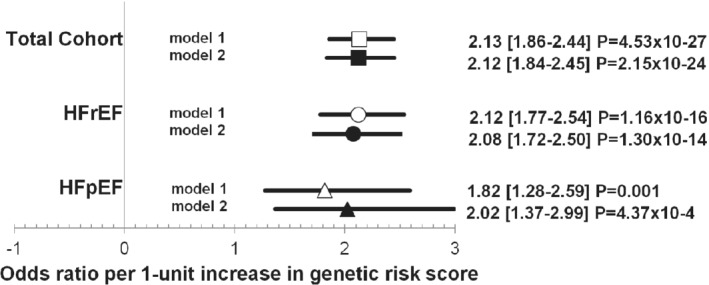
Genetic risk score and risk of atrial fibrillation prevalence. The bars signify the 95% confidence interval, the clear symbols represent results of model 1 and the solid symbols results of model 2. Squares indicate the total cohort, circles patients with heart failure with reduced ejection fraction (HFrEF), and triangles patients with heart failure with preserved ejection fraction (HFpEF). Model 1: adjusted for age, sex, and first 10 principal components of ancestry. Model 2: adjusted for age, height, weight, systolic and diastolic blood pressure, current smoking, hypertension, diabetes, myocardial infarction, and first 10 principal components of ancestry.

In the total cohort, the AF genetic risk score ranged between 4.62 to 8.29 with a median of 6.37. After multivariable adjustment, the odds ratio for AF presence was 2.12 per 1‐unit increase in genetic risk score (95% CI 1.84–2.45, *P* = 2.15 × 10^−24^) in the total BIOSTAT‐CHF cohort (*Figure* 2, Model 2). The odds ratio were 2.08 per 1‐unit increase in genetic risk score (95% CI 1.72–2.50, *P* = 1.30 × 10^−14^) in HFrEF and 2.02 per 1‐unit increase (95% CI 1.37–2.99, *P* = 4.37 × 10^−4^) in HFpEF, respectively.

**Figure 3:**
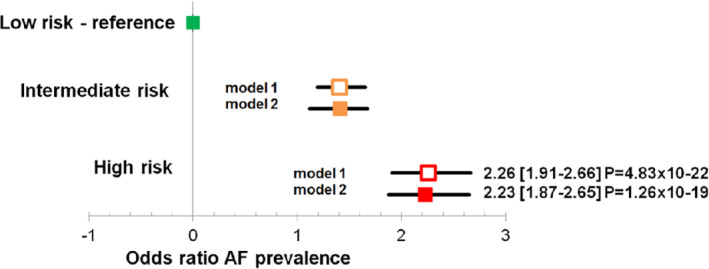
Increasing atrial fibrillation (AF) risk according to genetic risk score tertiles in the total cohort. The bars signify the 95% confidence interval, the clear symbols represent results of model 1 and the solid symbols results of model 2. Squares indicate the total cohort. Model 1: adjusted for age, sex, and first 10 principal components of ancestry. Model 2: adjusted for age, height, weight, systolic and diastolic blood pressure, current smoking, hypertension, diabetes, myocardial infarction, and first 10 principal components of ancestry.

There was no interaction between genetic risk score and heart failure type on AF prevalence (*P* = 0.99). We estimated odds ratios comparing individuals in genetic risk score tertiles (*Figure* 3). The odds ratio for AF prevalence increased with higher genetic risk score categories. For the total BIOSTAT‐CHF population, those in the highest tertile had 2.23 fold increased risk of AF compared to those in the lowest tertile (95% CI 1.87–2.65, *P* = 1.26 × 10^−19^).

### Heritability and atrial fibrillation prevalence classification models

Atrial fibrillation‐associated loci explain 22.9% of the overall AF SNP heritability (h_2_g) in our heart failure sample (*Table* 2).

**Table 2:** Proportion of heritability explained by atrial fibrillation loci

Study	AF‐loci h^2^_g_ observed (SE)	AF‐loci h^2^_g_ liability scale (SE)	Remaining genome h^2^_g_ observed (SE)	Remaining genome h^2^_g_ liability scale (SE)	Overall h^2^_g_ liability scale	Proportion explained (%)
97 AF loci	0.0557 (0.0297)	0.0876 (0.0468)	0.1873 (0.1135)	0.2947 (0.1786)	0.3823	22.92

AF, atrial fibrillation; h^2^_g_, single nucleotide polymorphism heritability; SE, standard error.

Proportion of AF single nucleotide polymorphism heritability explained by AF loci, defined as a 1 Mb region around sentinel variants.

The CHARGE‐AF risk model had an AUC of 0.699 (95% CI 0.682–0.716) for accurately classifying AF prevalence, and was better than the genetic risk score alone (AUC 0.606; 95% CI 0.588–0.624). Combining the AF genetic risk score with the CHARGE‐AF risk variables led to a model with an AUC of 0.721 (95% CI 0.704–0.737), a 2.2% increase over the CHARGE‐AF risk model alone (*Table* 3).

**Table 3:** Area under the receiver operating curves for atrial fibrillation risk models

Risk model	AUC (95% CI)	*P*‐value
CHARGE‐AF clinical risk score	0.699 (0.682–0.716)	<0.001
AF genetic risk score	0.606 (0.588–0.624)	<0.001
CHARGE‐AF clinical risk score + AF genetic risk score	0.721 (0.704–0.737)	<0.001

AF, atrial fibrillation; AUC, area under the receiver operating curve; CI, confidence interval.

### Genetic risk score and all‐cause mortality

During follow‐up, with a median of 656 days (interquartile range 448–872 days), 1062 patients died (28%). In the total cohort, the genetic risk score was not associated with an increased risk for all‐cause mortality after multivariable adjustment (HR 0.93, 95% CI 0.82–1.05, *P* = 0.22). Similar results were observed for the HFrEF (HR 0.92, 95% CI 0.78–1.08, *P* = 0.31) and HFpEF (HR 1.12, 95% CI 0.85–1.48, *P* = 0.44) subgroups. There was no interaction between heart failure subgroup and the genetic risk score on outcome (*P* = 0.63).

## Discussion

In 3759 heart failure patients of European ancestry, an AF genetic risk score, based on lead SNPs at 97 AF loci, was associated with a higher prevalence of AF after adjustment for clinical AF risk variables from the CHARGE‐AF risk model. We observed that 22.9% of variance in AF risk was attributable to additive genetic variation. Furthermore, addition of the AF genetic risk score to clinical risk factors improved risk model performance in classifying AF prevalence. The AF genetic risk score was not associated with all‐cause mortality. Our findings support and extend the prior observation that there is, at least, a partial genetic basis for AF in patients with HFrEF and HFpEF.^12^

### Genetic basis for atrial fibrillation in heart failure patients

Atrial fibrillation and heart failure frequently co‐exist, but direct causality has not been unequivocally proven. Additionally, the underlying mechanisms that lead to the development of AF in HFrEF and HFpEF and vice versa remain complex and not completely understood. Previously the *ZFHX3* gene was found to be associated with AF presence in a heart failure population.^12^ Our comprehensive AF genetic risk score of 97 SNPs, together with the estimation that 22.9% of the phenotypic variance is explained by additive genetic variation, provide evidence of a substantial contribution of genome‐wide variation to AF susceptibility in heart failure patients.

The genetic contribution to AF in our heart failure sample is less than what was previously observed in population based‐ and case‐referent AF‐GWAS studies, which also included a proportion of patients with heart failure (approximately 23% vs. 42%). Part of this portion of missing heritability may be caused by unidentified common genetic variants. Gene–environment interactions may also play a role, as genetic variants can also have associations with risk factors (pleiotropic effects). Heart failure patients have many risk factors including age, hypertension, diabetes, obesity, as well as valvular, ischaemic and non‐ischaemic structural heart disease.^10^, ^11^ On the other hand, increased cardiac filling pressures and consequently atrial stretch, cardiac fibrosis, dysregulation of intracellular calcium, and autonomic and neuroendocrine dysfunction in the setting of heart failure may evoke AF. It is possible that in the context of heart failure, with several concomitant risk factors, genetics may play a smaller role than in the general population.

It is hypothesized that AF in the presence of HFrEF is a marker of more advanced cardiac disease, with ventricular function deterioration and increased neurohormonal activation, while patients with AF and HFpEF share a more underlying substrate, albeit heterogeneous, with many shared risk factors.^10^, ^11^ A difference in the genetic contribution to AF in HFrEF or HFpEF is not evident from current results, as no interaction between genetic risk score and heart failure type was observed.

### Atrial fibrillation genetic risk score and all‐cause mortality

Previous analyses in BIOSTAT‐CHF have shown that worse cardiovascular outcomes were seen in heart failure patients with AF compared to sinus rhythm.^24^ Nevertheless, after multivariable adjustment, the AF genetic risk score was not associated with all‐cause mortality. However, a genetic risk score alone does not capture the clinical significance of AF presence in patients with an extensive cardiac substrate and other underlying risk factors. Additionally, current observations may be affected by survival bias.

### Implications

The clinical risk factor model alone outperformed the genetic risk score, this is to be expected since compared to clinical risk factors the effect size of genetic variants is small, even when combined in a polygenic risk score. Although the genetic risk score had moderate discriminatory accuracy, we demonstrated that a combined risk model, consisting of the AF genetic risk score with clinical AF risk factors as present in the CHARGE‐AF risk model, performed better than either risk model alone. But statistical significance does not automatically translate into clinical significance, and currently translation of genetics into clinical practice remains unresolved.

In the future, genetic profiling may provide insight into the mechanisms that underlie why some patients develop AF and others do not. The individual SNPs implicate genes that may reveal some of the mechanisms underlying AF (online supplementary *Figure S1*).^13^ Currently, most genes represent gene candidates at the loci, while the causal gene remains unknown. Experimental observations illustrate the pleiotropic nature of genes that are associated with this challenging arrhythmia and underscore the complexity of AF: so does *PITX2* encodes a transcription factor that plays a role in the formation of the pulmonary vein myocardium,^25^ does *TBX5* encodes transcription factors that are required for patterning and maturing of the cardiac conduction system in mice^26^ and have *KCNN3* and *SCN5A*, which both encode subunits of the potassium channel complex, been previously been linked to AF through candidate gene analyses and family‐based studies.^27^ More insights into the functional consequences of SNPs and genes is critical to identify potential therapeutic targets for this major health burden.^28^ However, whether the genetic proportion to AF risk has a meaningful contribution to clinical risk assessment warrants further investigation.

### Limitations

Current results, based on genetic data of 97 SNPs in 3759 patients from a well‐defined heart failure cohort, point towards a genetic basis for AF in the context of heart failure. Analyses were limited to European ancestry individuals, and the current heart failure sample had a higher percentage of men with only 30% of women, and a higher percentage of HFrEF than is typical in the community; the findings may not be completely generalizable to individuals of different ancestral backgrounds, regions, or the general heart failure population. Additionally, women and men generally have a different risk factor burden, which next to genetics and the underlying heart failure substrate, may be of different importance in the presence of concomitant heart failure and prevalent AF. Second, the genetic risk models were linear in nature with a single predictor variable and did not account for potential non‐additive genetic effects, interactions between genetic variants, or interactions between genetic variants and environmental factors. Therefore, all observations are vulnerable to the risk of residual confounding that may bias mentioned estimates. Thirdly, AF ascertainment was partially based on physician‐reported AF. This means that the percentage of AF is likely an underestimation since subclinical AF may have gone undetected. Fourthly, whether heart failure developed before the onset of AF, or AF before the onset of heart failure may be associated with a different genetic risk. Also the sequence in which the diseases develop can impact outcome. Unfortunately, we did not have information on the onset of AF and heart failure; therefore a temporal sequence of diagnoses was unknown, prohibiting time‐dependent analyses. AF occurrence during follow‐up was not systematically collected and therefore current analyses focus on baseline AF prevalence. Additionally, there was a lack of data on type and duration of AF, as well as applied therapies for AF. Fifthly, electro‐ and echocardiographic variables such as left atrial volume were omitted from the models since they were not available in a large proportion of patients. Additionally, these biomarkers will be influenced by both the underlying heart failure substrate as well as AF presence, duration, and severity. Covariates including LVEF, New York Heart Association class and NT‐proBNP will be confounded by AF itself as it inhibits adequate echocardiographic determination of ejection fraction, is associated with symptoms of dyspnoea, and will lead to an increase in NT‐proBNP levels. In line with the previous limitation, we did not adjust for heart failure severity in the multivariable models. We acknowledge that the CHARGE‐AF model application in heart failure was not ideal, albeit the best validated AF risk score. Sixthly, in determining SNP heritability we assessed variants with MAF ≥1%, and, therefore, the contribution of rare or loss‐of‐function variants to total AF variance was not assessed. Furthermore, the estimates for SNP heritability have large standard errors bringing a level of uncertainty to these estimates. Seventhly, we cannot attribute the AF risk variance to functional categories. It remains challenging to identify the causal gene at each locus since the AF‐associated SNPs predominantly fall within non‐coding portions of the genome. Additionally, the association of genes to functional groups is based on their affiliation to enriched gene sets that were identified in an *in silico* analysis. Lastly, establishing a heart failure cohort of sufficient size is complex, and the current study is underpowered to study individual SNPs or perform extensive subgroup analyses. Larger studies, powered for outcomes, are warranted to investigate the genetic contribution to incident AF in heart failure populations, both HFrEF and HFpEF. Further efforts are needed to uncover the functional consequence of SNPs and genes at each locus on AF risk in patients with incident heart failure.

## Conclusion

The AF genetic risk score was associated with increased AF prevalence in heart failure patients with reduced and preserved ejection fraction. Genetic variation accounted for 22.9% overall AF SNP heritability. Addition of the AF genetic risk score to clinical risk factors improved risk model performance in classifying AF prevalence. Efforts are warranted to consider the role and mechanisms of genetic susceptibility of AF risk in heart failure patients.

### Funding

This work was supported by the Netherlands Cardiovascular Research Initiative: an initiative with support of the Dutch Heart Foundation; Renal Connection to microvascular disease and heart failure with preserved ejection fraction [CVON2014‐11 RECONNECT] and a grant from the European Commission [FP7‐242209‐BIOSTAT‐CHF]. C.P.N. and N.J.S. are funded by the British Heart Foundation.

**Conflict of interest:** C.R. is supported by a grant from Bayer AG to the Broad Institute focused on the development of therapeutics for cardiovascular disease. C.S.P.L. reports grants from National Medical Research Council of Singapore; grants and personal fees from Boston Scientific, Bayer, Roche Diagnostic, Vifor Pharma; grants from Medtronic; personal fees from AstraZeneca, Novartis, Amgen, Merck, Janssen Research & Development LLC, Menarini, Boehringer Ingelheim, Abbott Diagnostics, Corvia and Stealth BioTherapeutics, outside the submitted work. C.C.L. reports grants and other from AstraZeneca; grants from Amgen, Novartis; other from MSD and Servier, during the conduct of the study. L.L.N. reports grants from European Union FP7 programme, and John & Lucille Van Geest Foundation, during the conduct of the study. M.M. reports grants from European Community, during the conduct of the study; personal fees from Bayer, Novartis and Servier, outside the submitted work. S.A. reports grants and personal fees from Vifor Int; personal fees from Bayer, Boehringer Ingelheim, Novartis, Servier, Respicardia, Impulse Dynamics; grants from Abbott Vascular, outside the submitted work. D.J.V.V. reports board membership fees/travel expenses from Johnson & Johnson, Novartis. E.J.B. reports grants from NHLBI (R01HL128914; 2R01 HL092577) and American Heart Association (18SFRN34110082). A.A.V. reports grants from European Commission, during the conduct of the study; personal fees from Amgen, Boehringer Ingelheim, AstraZeneca, Bayer, Cytokinetics, GSK, Myokardia, Novartis, Servier; grants and personal fees from Roche Diagnostics, outside the submitted work. The other authors have nothing to disclose.

## Supporting information


**Table S1.** SNPs and weights used in the AF genetic risk score.
**Figure S1.** Venn diagram.

## References

[1] Maisel WH , Stevenson LW . Atrial fibrillation in heart failure: epidemiology, pathophysiology, and rationale for therapy. Am J Cardiol 2003;91:2D–8D. 10.1016/s0002-9149(02)03373-8 12670636

[2] Sartipy U , Dahlstrom U , Fu M , Lund LH . Atrial fibrillation in heart failure with preserved, mid‐range, and reduced ejection fraction. JACC Heart Fail 2017;5:565–574. 28711451 10.1016/j.jchf.2017.05.001

[3] Zafrir B , Lund LH , Laroche C , Ruschitzka F , Crespo‐Leiro MG , Coats AJ , Anker SD , Filippatos G , Seferovic PM , Maggioni AP , De Mora Martin M , Polonski L , Silva‐Cardoso J , Amir O ; ESC‐HFA HF Long‐Term Registry Investigators. Prognostic implications of atrial fibrillation in heart failure with reduced, mid‐range, and preserved ejection fraction: a report from 14 964 patients in the European Society of Cardiology Heart Failure Long‐Term Registry. Eur Heart J 2018;39:4277–4284. 30325423 10.1093/eurheartj/ehy626

[4] Staerk L , Wang B , Preis SR , Larson MG , Lubitz SA , Ellinor PT , McManus DD , Ko D , Weng LC , Lunetta KL , Frost L , Benjamin EJ , Trinquart L . Lifetime risk of atrial fibrillation according to optimal, borderline, or elevated levels of risk factors: cohort study based on longitudinal data from the Framingham Heart Study. BMJ 2018;361:k1453. 29699974 10.1136/bmj.k1453 PMC5917175

[5] Weng LC , Preis SR , Hulme OL , Larson MG , Choi SH , Wang B , Trinquart L , McManus DD , Staerk L , Lin H , Lunetta KL , Ellinor PT , Benjamin EJ , Lubitz SA . Genetic predisposition, clinical risk factor burden, and lifetime risk of atrial fibrillation. Circulation 2018;137:1027–1038. 29129827 10.1161/CIRCULATIONAHA.117.031431 PMC5840011

[6] Magnussen C , Niiranen TJ , Ojeda FM , Gianfagna F , Blankenberg S , Njolstad I , Vartiainen E , Sans S , Pasterkamp G , Hughes M , Costanzo S , Donati MB , Jousilahti P , Linneberg A , Palosaari T , de Gaetano G , Bobak M , den Ruijter HM , Mathiesen E , Jorgensen T , Soderberg S , Kuulasmaa K , Zeller T , Iacoviello L , Salomaa V , Schnabel RB ; BiomarCaRE Consortium . Sex differences and similarities in atrial fibrillation epidemiology, risk factors, and mortality in community cohorts: results from the BiomarCaRE Consortium (Biomarker for Cardiovascular Risk Assessment in Europe). Circulation 2017;136:1588–1597. 29038167 10.1161/CIRCULATIONAHA.117.028981 PMC5657474

[7] Benjamin EJ , Muntner P , Alonso A , Bittencourt MS , Callaway CW , Carson AP , Chamberlain AM , Chang AR , Cheng S , Das SR , Delling FN , Djousse L , Elkind MSV , Ferguson JF , Fornage M , Jordan LC , Khan SS , Kissela BM , Knutson KL , Kwan TW , Lackland DT , Lewis TT , Lichtman JH , Longenecker CT , Loop MS , Lutsey PL , Martin SS , Matsushita K , Moran AE , Mussolino ME , O'Flaherty M , Pandey A , Perak AM , Rosamond WD , Roth GA , Sampson UKA , Satou GM , Schroeder EB , Shah SH , Spartano NL , Stokes A , Tirschwell DL , Tsao CW , Turakhia MP , VanWagner LB , Wilkins JT , Wong SS , Virani SS . American Heart Association Council on Epidemiology and Prevention Statistics Committee and Stroke Statistics Subcommittee. Heart disease and stroke statistics – 2019 update: a report from the American Heart Association. Circulation 2019;139:e56–e528. 30700139 10.1161/CIR.0000000000000659

[8] Kotecha D , Chudasama R , Lane DA , Kirchhof P , Lip GY . Atrial fibrillation and heart failure due to reduced versus preserved ejection fraction: a systematic review and meta‐analysis of death and adverse outcomes. Int J Cardiol 2016;203:660–666. 26580351 10.1016/j.ijcard.2015.10.220

[9] Santhanakrishnan R , Wang N , Larson MG , Magnani JW , McManus DD , Lubitz SA , Ellinor PT , Cheng S , Vasan RS , Lee DS , Wang TJ , Levy D , Benjamin EJ , Ho JE . Atrial fibrillation begets heart failure and vice versa: temporal associations and differences in preserved versus reduced ejection fraction. Circulation 2016;133:484–492. 26746177 10.1161/CIRCULATIONAHA.115.018614 PMC4738087

[10] Kotecha D , Piccini JP . Atrial fibrillation in heart failure: what should we do? Eur Heart J 2015;36:3250–3257. 26419625 10.1093/eurheartj/ehv513 PMC4670966

[11] Lee Park K , Anter E . Atrial fibrillation and heart failure: a review of the intersection of two cardiac epidemics. J Atr Fibrillation 2013;6:751. 28496849 10.4022/jafib.751 PMC5153058

[12] Smith JG , Melander O , Sjogren M , Hedblad B , Engstrom G , Newton‐Cheh C , Platonov PG . Genetic polymorphisms confer risk of atrial fibrillation in patients with heart failure: a population‐based study. Eur J Heart Fail 2013;15:250–257. 23132824 10.1093/eurjhf/hfs176 PMC3576900

[13] Roselli C , Chaffin MD , Weng LC , Aeschbacher S , Ahlberg G , Albert CM , Almgren P , Alonso A , Anderson CD , Aragam KG , Arking DE , Barnard J , Bartz TM , Benjamin EJ , Bihlmeyer NA , Bis JC , Bloom HL , Boerwinkle E , Bottinger EB , Brody JA , Calkins H , Campbell A , Cappola TP , Carlquist J , Chasman DI , Chen LY , Chen YI , Choi EK , Choi SH , Christophersen IE , Chung MK , Cole JW , Conen D , Cook J , Crijns HJ , Cutler MJ , Damrauer SM , Daniels BR , Darbar D , Delgado G , Denny JC , Dichgans M , Dorr M , Dudink EA , Dudley SC , Esa N , Esko T , Eskola M , Fatkin D , Felix SB , Ford I , Franco OH , Geelhoed B , Grewal RP , Gudnason V , Guo X , Gupta N , Gustafsson S , Gutmann R , Hamsten A , Harris TB , Hayward C , Heckbert SR , Hernesniemi J , Hocking LJ , Hofman A , Horimoto ARVR , Huang J , Huang PL , Huffman J , Ingelsson E , Ipek EG , Ito K , Jimenez‐Conde J , Johnson R , Jukema JW , Kaab S , Kahonen M , Kamatani Y , Kane JP , Kastrati A , Kathiresan S , Katschnig‐Winter P , Kavousi M , Kessler T , Kietselaer BL , Kirchhof P , Kleber ME , Knight S , Krieger JE , Kubo M , Launer LJ , Laurikka J , Lehtimaki T , Leineweber K , Lemaitre RN , Li M , Lim HE , Lin HJ , Lin H , Lind L , Lindgren CM , Lokki ML , London B , Loos RJF , Low SK , Lu Y , Lyytikainen LP , Macfarlane PW , Magnusson PK , Mahajan A , Malik R , Mansur AJ , Marcus GM , Margolin L , Margulies KB , Marz W , McManus DD , Melander O , Mohanty S , Montgomery JA , Morley MP , Morris AP , Muller‐Nurasyid M , Natale A , Nazarian S , Neumann B , Newton‐Cheh C , Niemeijer MN , Nikus K , Nilsson P , Noordam R , Oellers H , Olesen MS , Orho‐Melander M , Padmanabhan S , Pak HN , Pare G , Pedersen NL , Pera J , Pereira A , Porteous D , Psaty BM , Pulit SL , Pullinger CR , Rader DJ , Refsgaard L , Ribases M , Ridker PM , Rienstra M , Risch L , Roden DM , Rosand J , Rosenberg MA , Rost N , Rotter JI , Saba S , Sandhu RK , Schnabel RB , Schramm K , Schunkert H , Schurman C , Scott SA , Seppala I , Shaffer C , Shah S , Shalaby AA , Shim J , Shoemaker MB , Siland JE , Sinisalo J , Sinner MF , Slowik A , Smith AV , Smith BH , Smith JG , Smith JD , Smith NL , Soliman EZ , Sotoodehnia N , Stricker BH , Sun A , Sun H , Svendsen JH , Tanaka T , Tanriverdi K , Taylor KD , Teder‐Laving M , Teumer A , Theriault S , Trompet S , Tucker NR , Tveit A , Uitterlinden AG , Van Der Harst P , Van Gelder IC , Van Wagoner DR , Verweij N , Vlachopoulou E , Volker U , Wang B , Weeke PE , Weijs B , Weiss R , Weiss S , Wells QS , Wiggins KL , Wong JA , Woo D , Worrall BB , Yang PS , Yao J , Yoneda ZT , Zeller T , Zeng L , Lubitz SA , Lunetta KL , Ellinor PT . Multi‐ethnic genome‐wide association study for atrial fibrillation. Nat Genet 2018;50:1225–1233. 29892015 10.1038/s41588-018-0133-9 PMC6136836

[14] Voors AA , Anker SD , Cleland JG , Dickstein K , Filippatos G , van der Harst P , Hillege HL , Lang CC , Ter Maaten JM , Ng L , Ponikowski P , Samani NJ , van Veldhuisen DJ , Zannad F , Zwinderman AH , Metra M . A systems BIOlogy Study to TAilored Treatment in Chronic Heart Failure: rationale, design, and baseline characteristics of BIOSTAT‐CHF. Eur J Heart Fail 2016;18:716–726. 27126231 10.1002/ejhf.531

[15] Delaneau O , Zagury JF , Marchini J . Improved whole‐chromosome phasing for disease and population genetic studies. Nat Methods 2013;10:5–6. 23269371 10.1038/nmeth.2307

[16] Howie BN , Donnelly P , Marchini J . A flexible and accurate genotype imputation method for the next generation of genome‐wide association studies. PLoS Genet 2009;5:e1000529. 19543373 10.1371/journal.pgen.1000529 PMC2689936

[17] Sudmant PH , Rausch T , Gardner EJ , Handsaker RE , Abyzov A , Huddleston J , Zhang Y , Ye K , Jun G , Fritz MH , Konkel MK , Malhotra A , Stutz AM , Shi X , Casale FP , Chen J , Hormozdiari F , Dayama G , Chen K , Malig M , Chaisson MJ , Walter K , Meiers S , Kashin S , Garrison E , Auton A , Lam HY , Mu XJ , Alkan C , Antaki D , Bae T , Cerveira E , Chines P , Chong Z , Clarke L , Dal E , Ding L , Emery S , Fan X , Gujral M , Kahveci F , Kidd JM , Kong Y , Lameijer EW , McCarthy S , Flicek P , Gibbs RA , Marth G , Mason CE , Menelaou A , Muzny DM , Nelson BJ , Noor A , Parrish NF , Pendleton M , Quitadamo A , Raeder B , Schadt EE , Romanovitch M , Schlattl A , Sebra R , Shabalin AA , Untergasser A , Walker JA , Wang M , Yu F , Zhang C , Zhang J , Zheng‐Bradley X , Zhou W , Zichner T , Sebat J , Batzer MA , McCarroll SA , Mills RE , Gerstein MB , Bashir A , Stegle O , Devine SE , Lee C , Eichler EE , Korbel JO ; 1000 Genomes Project Consortium . An integrated map of structural variation in 2,504 human genomes. Nature 2015;526:75–81. 26432246 10.1038/nature15394 PMC4617611

[18] Chang CC , Chow CC , Tellier LC , Vattikuti S , Purcell SM , Lee JJ . Second‐generation PLINK: rising to the challenge of larger and richer datasets. Gigascience 2015;4:7. 25722852 10.1186/s13742-015-0047-8 PMC4342193

[19] Loh PR , Bhatia G , Gusev A , Finucane HK , Bulik‐Sullivan BK , Pollack SJ , de Candia TR , Lee SH , Wray NR , Kendler KS , O'Donovan MC , Neale BM , Patterson N , Price AL ; Schizophrenia Working Group of Psychiatric Genomics Consortium . Contrasting genetic architectures of schizophrenia and other complex diseases using fast variance‐components analysis. Nat Genet 2015;47:1385–1392. 26523775 10.1038/ng.3431 PMC4666835

[20] QCTOOL: a command‐line utility program for manipulation and quality control of gwas datasets and other genome‐wide data. https://www.well.ox.ac.uk/∼gav/qctool_v2/ [accessed 16 December 2019].

[21] Lee SH , Wray NR , Goddard ME , Visscher PM . Estimating missing heritability for disease from genome‐wide association studies. Am J Hum Genet 2011;88:294–305. 21376301 10.1016/j.ajhg.2011.02.002 PMC3059431

[22] Alonso A , Krijthe BP , Aspelund T , Stepas KA , Pencina MJ , Moser CB , Sinner MF , Sotoodehnia N , Fontes JD , Janssens AC , Kronmal RA , Magnani JW , Witteman JC , Chamberlain AM , Lubitz SA , Schnabel RB , Agarwal SK , McManus DD , Ellinor PT , Larson MG , Burke GL , Launer LJ , Hofman A , Levy D , Gottdiener JS , Kaab S , Couper D , Harris TB , Soliman EZ , Stricker BH , Gudnason V , Heckbert SR , Benjamin EJ . Simple risk model predicts incidence of atrial fibrillation in a racially and geographically diverse population: the CHARGE‐AF consortium. J Am Heart Assoc 2013;2:e000102. 23537808 10.1161/JAHA.112.000102 PMC3647274

[23] Robin X , Turck N , Hainard A , Tiberti N , Lisacek F , Sanchez JC , Muller M . pROC: an open‐source package for R and S+ to analyze and compare ROC curves. BMC Bioinformatics 2011;12:77. 21414208 10.1186/1471-2105-12-77 PMC3068975

[24] Santema BT , Kloosterman M , Van Gelder IC , Mordi I , Lang CC , Lam CS , Anker SD , Cleland JG , Dickstein K , Filippatos G , Van der Harst P , Hillege HL , Ter Maaten JM , Metra M , Ng LL , Ponikowski P , Samani NJ , Van Veldhuisen DJ , Zwinderman AH , Zannad F , Damman K , Van der Meer P , Rienstra M , Voors AA . Comparing biomarker profiles of patients with heart failure: atrial fibrillation vs. sinus rhythm and reduced vs. preserved ejection fraction. Eur Heart J 2018;39:3867–3875. 30137304 10.1093/eurheartj/ehy421

[25] Mommersteeg MT , Brown NA , Prall OW , de Gier‐de Vries C , Harvey RP , Moorman AF , Christoffels VM . Pitx2c and Nkx2‐5 are required for the formation and identity of the pulmonary myocardium. Circ Res 2007;101:902–909. 17823370 10.1161/CIRCRESAHA.107.161182

[26] Arnolds DE , Liu F , Fahrenbach JP , Kim GH , Schillinger KJ , Smemo S , McNally EM , Nobrega MA , Patel VV , Moskowitz IP . TBX5 drives Scn5a expression to regulate cardiac conduction system function. J Clin Invest 2012;122:2509–2518. 22728936 10.1172/JCI62617 PMC3386825

[27] Ellinor PT , Lunetta KL , Glazer NL , Pfeufer A , Alonso A , Chung MK , Sinner MF , de Bakker PI , Mueller M , Lubitz SA , Fox E , Darbar D , Smith NL , Smith JD , Schnabel RB , Soliman EZ , Rice KM , Van Wagoner DR , Beckmann BM , van Noord C , Wang K , Ehret GB , Rotter JI , Hazen SL , Steinbeck G , Smith AV , Launer LJ , Harris TB , Makino S , Nelis M , Milan DJ , Perz S , Esko T , Kottgen A , Moebus S , Newton‐Cheh C , Li M , Mohlenkamp S , Wang TJ , Kao WH , Vasan RS , Nothen MM , MacRae CA , Stricker BH , Hofman A , Uitterlinden AG , Levy D , Boerwinkle E , Metspalu A , Topol EJ , Chakravarti A , Gudnason V , Psaty BM , Roden DM , Meitinger T , Wichmann HE , Witteman JC , Barnard J , Arking DE , Benjamin EJ , Heckbert SR , Kaab S . Common variants in KCNN3 are associated with lone atrial fibrillation. Nat Genet 2010;42:240–244. 20173747 10.1038/ng.537 PMC2871387

[28] Lubitz SA , Rienstra M . Genetic susceptibility to atrial fibrillation: does heart failure change our perspective? Eur J Heart Fail 2013;15:244–246. 23315103 10.1093/eurjhf/hft005

