# Supplementary material for: Genetic risk and atrial fibrillation in patients with heart failure
Source: Eur J Heart Fail. 2020 Jan 9;22(3):519–27. doi: 10.1002/ejhf.1735 (PMC7319410; doi:10.1002/ejhf.1735)
Supplement: Supplementary file 1 — Table S1. SNPs and weights used in the AF genetic risk score. Figure S1. Venn diagram. [file EJHF-22-519-s001.pdf]

SUPPLEMENTAL MATERIAL

Supplemental Table 1. SNPs and weights used in the AF genetic risk score

Rsid	Chr	BP (hg19)	Risk Allele	Weight
rs187585530	1	10167425	A	0.4390
rs880315	1	10796866	C	0.0437
rs146518726	1	51535039	A	0.1617
rs12044963	1	112392360	T	0.0795
rs4484922	1	116310818	G	0.0630
rs79187193	1	147255831	G	0.1116
rs11264280	1	154862952	T	0.1270
rs72700114	1	170193825	C	0.2026
rs608930	1	170617306	G	0.0968
rs10753933	1	203026214	T	0.0743
rs4951261	1	205717823	C	0.0441
rs6546620	2	26159940	C	0.0708
rs6742276	2	61768745	A	0.0485
rs2540949	2	65284231	A	0.0752
rs10165883	2	70117015	C	0.0642
rs72926475	2	86594487	G	0.0708
rs56181519	2	175555714	C	0.0778
rs35504893	2	179421294	T	0.0900
rs295114	2	201195602	C	0.0676
rs6810325	3	12840934	C	0.0747
rs6790396	3	38771925	G	0.0636
rs2306272	3	66434643	C	0.0512
rs7632427	3	89534377	T	0.0460
rs17490701	3	111587879	G	0.0700
rs4855075	3	179170494	T	0.0604
rs3822259	4	10118745	T	0.0463
rs3960788	4	103915618	C	0.0507
rs2129977	4	111712432	A	0.4016
rs55754224	4	114428714	T	0.0477
rs10213171	4	148937537	G	0.1041
rs10520260	4	174447349	A	0.0539
rs716845	5	113736416	A	0.0594
rs34750263	5	137434172	T	0.0873
rs174048	5	142650404	C	0.0665
rs6882776	5	172664163	G	0.0600
rs73366713	6	16415751	G	0.1052
rs34969716	6	18210109	A	0.0875
rs3176326	6	36647289	G	0.0599
rs17079881	6	118566187	G	0.0851
rs13191450	6	122392136	A	0.0704

Rsid	Chr	BP (hg19)	Risk Allele	Weight
rs117984853	6	149399100	T	0.1132
rs55734480	7	14372009	A	0.0504
rs6462078	7	28413187	A	0.0580
rs74910854	7	74110705	G	0.0942
rs11773884	7	92285123	A	0.0486
rs62483627	7	106856002	A	0.0489
rs11773845	7	116191301	A	0.1162
rs7789146	7	150661409	G	0.0571
rs7508	8	17913970	A	0.0720
rs7846485	8	21803735	C	0.0872
rs62521286	8	124551975	G	0.1224
rs35006907	8	125859817	A	0.0454
rs6993266	8	141762659	A	0.0443
rs4977397	9	20235004	A	0.0432
rs4385527	9	97648587	A	0.0920
rs4743034	9	109632353	A	0.0490
rs10760361	9	127178266	G	0.0434
rs7919685	10	65315800	G	0.0579
rs60212594	10	75414344	G	0.1097
rs11001667	10	77935345	G	0.0619
rs1044258	10	103605714	T	0.0463
rs11598047	10	105342672	G	0.1533
rs1822273	11	20010513	G	0.0683
rs949078	11	121629007	C	0.0534
rs76097649	11	128764570	A	0.1264
rs10842383	12	24771967	C	0.1088
rs113819537	12	26348429	C	0.0490
rs12809354	12	32978437	C	0.0810
rs7978685	12	57103154	T	0.0547
rs35349325	12	70097464	T	0.0524
rs11180703	12	76223817	G	0.0457
rs883079	12	114793240	T	0.1196
rs12810346	12	115091017	T	0.0658
rs12298484	12	124418674	C	0.0455
rs9580438	13	23373406	C	0.0568
rs28631169	14	23888183	T	0.0700
rs2145587	14	32981484	A	0.0754
rs73241997	14	35173775	T	0.0720
rs2738413	14	64679960	A	0.0807
rs10873299	14	77426711	A	0.0483
rs62011291	15	63800013	G	0.0519
rs12591736	15	70454139	G	0.0606
rs74022964	15	73677264	T	0.1059

Rsid	Chr	BP (hg19)	Risk Allele	Weight
rs12908004	15	80676925	G	0.0753
rs12908437	15	99287375	T	0.0468
rs2286466	16	2014283	G	0.0718
rs2359171	16	73053022	A	0.1884
rs8073937	17	7435040	G	0.0504
rs72811294	17	12618680	G	0.0667
rs242557	17	44019712	G	0.0439
rs76774446	17	45046368	A	0.0654
rs7219869	17	68337185	G	0.0460
rs9953366	18	46474192	C	0.0504
rs2145274	20	6572014	A	0.1015
rs7269123	20	61157939	C	0.0443
rs2834618	21	36119111	T	0.1096
rs361834*	22	18597404	G	0.0470

* proxy for rs465276 (r^2=0.91)

Supplemental Figure 1. Venn diagram

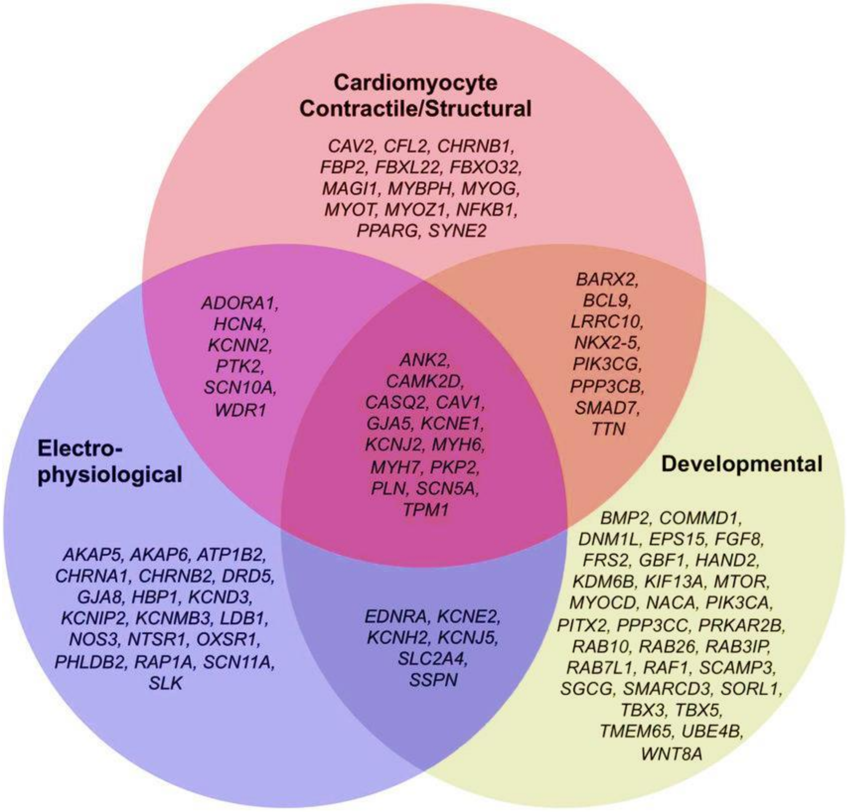

The Venn diagram shows overlap between the gene(s) closest to the 97 SNPs and functional categories based on their corresponding gene sets. Genes were manually assigned to one or more functional groups based on their affiliation to gene sets. Adapted from Roselli et al. *Nat Genetics* 2018 with permission.
